# Pesticide and Liver Biomarkers Among Ecuadorian Adolescents and Adults Living in Agricultural Settings

**DOI:** 10.3390/toxics13080685

**Published:** 2025-08-18

**Authors:** Priyanka Mehta, Rajendra P. Parajuli, Briana N. C. Chronister, Kun Yang, Dana B. Barr, Xin M. Tu, Dolores Lopez-Paredes, Jose R. Suarez-Lopez

**Affiliations:** 1 Herbert Wertheim School of Public Health and Human Longevity Science, University of California San Diego (UCSD), La Jolla, CA 92093, USArparajuli@health.ucsd.edu (R.P.P.); bnchronister@health.ucsd.edu (B.N.C.C.); yangkun123332@gmail.com (K.Y.); x2tu@health.ucsd.edu (X.M.T.); 2 Gangarosa Department of Environmental Health, Rollins School of Public Health, Emory University, Atlanta, GA 30322, USA; dbbarr@emory.edu; 3 Fundación Cimas del Ecuador (CIMAS), De los Olivos E14-226 y, Quito 170124, Ecuador

**Keywords:** pesticide, insecticide and herbicide urinary metabolite, liver enzymes and biomarkers, adolescents, young adult, ESPINA study, Ecuador

## Abstract

**Background:** Experimental studies suggest that some insecticides, fungicides, and herbicides can result in liver cell death, but population-based evidence is lacking. We investigated associations between urinary pesticide metabolites and liver biomarkers among adolescents and adults in an Ecuadorian agricultural area. **Methods:** We examined participants in 2016 (N = 528, 11–17 years) and 2022 (N = 505, 17–24 years). Plasma alanine aminotransferase (ALT), aspartate aminotransferase, soluble cytokeratin-18, and erythrocytic acetylcholinesterase were measured. Urinary biomarkers included four organophosphates, six neonicotinoids, three pyrethroids, two herbicides, and two fungicides. Generalized estimating equation (GEE) models examined associations and introduced sex and age interaction terms and quadratic terms. Quantile g-computation evaluated the effects of pesticide mixtures. **Results:** No significant associations were observed between pesticide biomarkers and liver biomarkers in longitudinal or cross-sectional analyses. A curvilinear association was found between 3-phenoxybenzoic acid (3-PBA; pyrethroid) and ALT (β_quadratic_ = −0.35, 95% CI: [−0.67, −0.04]) in 2016, but not in 2022. Sex modified the associations of 3-PBA with AST, ALT, and CK18-M65 in adolescents (2016), with non-significant positive associations observed in males and non-significant negative associations observed in females. No pesticide mixture effects were observed. **Conclusions:** Urinary biomarkers of various insecticides, herbicides, fungicides, and their mixtures were not associated with liver biomarkers among adolescents and young adults in agricultural settings. These largely null findings, consistent across time points, suggest background-level exposures in these settings possibly do not harm liver health in this population, though effects at higher exposures cannot be ruled out.

## 1. Introduction

Pesticides are a major concern worldwide since their application puts humans at risk of exposure, either directly or indirectly through their food or environment [1]. Pesticides can enter the body through inhalation, dermal absorption, and ingestion [2,3]. The increased use of these pesticides has raised concerns about potential adverse effects on human health and the subsequent burdens on the healthcare system [4]. In particular, liver diseases are a global health concern, resulting in approximately two million deaths per year (i.e., 4% of all deaths/one out of every twenty-five deaths worldwide) [5].

Numerous studies have documented the harmful effects of pesticides on the liver in animal studies. A recent study showed that long-term administration of para-nitrophenol (PNP, a metabolite of the organophosphates parathion and methyl parathion) caused hepatotoxicity (i.e., a significant increase in aspartate aminotransferase (AST) levels, apoptosis, and hepatocyte proliferation) in Japanese quail and that PNP had profound toxic effects on the liver at the cellular level [6]. Similarly, Baconi et al. (2013) carried out a repeated-dose study to investigate the toxicity of malathion and diazinon in Wistar rats and reported that repeated exposure, at subclinical doses, to malathion dicarboxylic acid (MDA; the major specific malathion metabolite) caused moderate-to-severe biochemical, histopathological, and immune alterations (i.e., vasodilatation, microvacuoles, and granular dystrophy) in the liver [7]. A study by Al-Othman et al. (2012) showed that oral administration of malathion in adult male rats induced severe hepatic injury, as shown by increasing levels of alanine aminotransferase (ALT) and AST [8]. Animals exposed to pesticides, such as organophosphates (OPs), methyl carbamates, and pyrethroids, exhibit changes in liver chemistry parameters [9]. Recent studies on animals have revealed that glyphosate and the bis-dithiocarbamate fungicide metabolites ethylene thiourea (ETU) and propylene thiourea (PTU) can affect the liver through mechanisms involving inflammation and oxidative stress [10,11,12].

A growing body of epidemiological studies that evaluate the effects of pesticides on the liver is also emerging. For example, Huang et al. (2016) assessed 224 Chinese farmers to identify associations between exposure to OPs, organosulfurs (a class of pesticides that includes sulfur-containing fungicides, including bis-dithiocarbamates), organonitrogens (which include certain herbicides and fungicides containing nitrogen groups), and pyrethroids with blood liver health markers. Pesticide exposure was estimated as the amount (in kilograms) of pesticidal active ingredients applied by farmers [13]. Short-term exposure to both OPs and organosulfurs was associated with increased levels of ALT and AST (*p*-value < 0.01) [13]. Similarly, a recent study in southern Brazil conducted by Bernieri et al. (2021) evaluated hepatic parameters [ALT, AST, and gamma-glutamyl transferase (GGT)] during high- and low-pesticide-spray periods among adult soybean farmers [14]. They reported a statistically significant increase in AST in high-pesticide-spray periods compared with low-pesticide-spray periods; however, ALT and GGT did not differ between the two periods [14]. More recent studies using urinary pesticide metabolite biomarkers have added mechanistic insight into pesticide-induced hepatotoxicity. For instance, Ismail et al. (2021) found that urinary 3,5,6-trichloro-2-pyridinol (TCPy) (a chlorpyrifos metabolite) was significantly associated with increased ALT and AST in 602 Egyptian adolescents [15]. In China, Jia et al. (2025) reported that urinary TCPy, PNP, and 3-PBA were significantly linked to lipid metabolism disruption, with ALT and AST acting as mediators in a cohort of 1858 adolescents and young adults [16]. In adults, multiple studies, including Li et al. (2022) and Dong et al. (2024), identified significant associations between urinary dialkyl phosphate (DAP) metabolites (from OPs) and elevated ALT/AST among U.S. adults using the National Health and Nutrition Examination Survey (NHANES) data [17,18]. Nguyen and Kim (2022) similarly observed that higher levels of urinary pesticide metabolites were linked with increased ALT and AST in 2130 Korean adults, particularly among older males [19]. In contrast, Godbole et al. (2024) found no significant associations between urinary neonicotinoid metabolites and liver enzymes (ALT [β = 0.71], AST [β = 0.31]) in 1695 U.S. adults, largely attributed to the low detection rates of these compounds (<40%) [20]. Together, these studies link pesticides to liver dysfunction, although more longitudinal and mechanistic studies are needed to confirm causality and evaluate low-detection pesticide classes.

The liver and kidneys are vulnerable to damage by pesticides [9,14,21]. Pesticides induce stress and damage the liver by causing lipid peroxidation, leading to damage to hepatocytes and resulting in the production of ALT and AST in the body due to cell membrane damage and leakage [14,22]. Cytokeratin 18 (CK18) is a main intermediate filament protein in hepatocytes that is released into the blood upon the initiation of cell death [23]. Apoptosis (i.e., programmed cell death) causes the release of caspase-cleaved CK18 and induces the production of M30, a monoclonal antibody that recognizes the breakdown neuroepitope of CK18, whereas necrosis (i.e., accidental, uncontrolled cell death) releases CK18 and induces the production of M65, a monoclonal antibody that recognizes all CK18 fragments. Hence, the CK18 (M30) fraction is an indicator of hepatocyte apoptosis, and CK18 (M65) is an indicator of hepatocyte necrosis [24,25,26]. ALT and AST, the most frequently utilized and specific indicators of hepatocellular necrosis [27,28], are considered biomarkers for acute hepatotoxicity. Additionally, long-term exposure to these harmful chemicals can also lead to severe liver damage [29].

Families that are primarily engaged in farming or live in rural communities are at greater risk of exposure to pesticides [14,30]. In rural settings, children are exposed to pesticides in areas where chemicals are applied, maintained, or contaminated by work equipment, food, groundwater, and their parents’ clothing. Children, a group that is the most vulnerable to the health effects of pesticide exposure [31,32], often eat and drink more relative to their body weight than adults do, which can lead to a higher dose of pesticide deposit per pound of body weight [33,34].

Studies on the impacts of pesticide exposure on liver health among children and adolescents are lacking. Thus, this study aimed to determine whether pesticide exposure biomarkers are associated with liver biomarkers (ALT, AST, CK18 M30, and CK18 M65) when assessed prospectively from adolescence into young adulthood. Based on prior findings, we hypothesized that higher pesticide exposure was associated with elevated liver biomarkers.

## 2. Materials and Methods

### 2.1. Study Area

The Secondary Exposures to Pesticides among Children, Adolescents and Adults (ESPINA) study was established in 2008 in Pedro Moncayo, Ecuador, to examine the associations between pesticide exposure and child development [35]. Pedro Moncayo County is located in the Ecuadorian Andes at an average altitude of 2952 m. The floriculture industry is vital to the economy of this county, employing 21% of all adults in the county [35]. It has substantial floricultural activity [36], with greenhouse floricultural crop land comprising 4.47% of the geographic area (1495 hectares). The Ecuadorian floriculture industry uses a wide range of pesticides, including fungicides, herbicides, and insecticides, which are sprayed by workers using hand sprayers [37], with organophosphates and carbamates accounting for 71% of pesticide poisonings in Ecuador [38].

### 2.2. Study Sample

In 2008, approximately 73% of the study participants were recruited through public announcements inviting individuals who had taken part in the 2004 Survey of Access and Demand for Health Services in Pedro Moncayo County (SADHS-PM). This earlier survey, conducted by Fundación Cimas del Ecuador in collaboration with local communities, was designed to be representative of the county’s population. Additional details regarding the 2008 participant recruitment process have been published previously [35].

In 2016, a total of 554 adolescents aged 12 to 17 years participated in two assessment periods: 330 were evaluated in April and 535 between July and October, with 311 individuals participating in both time frames. Of these, 238 had also taken part in the 2008 assessment, while 316 were newly enrolled. Recruitment of new participants was carried out using the System of Local and Community Information (SILC), developed by Fundación Cimas del Ecuador, which incorporated data from the 2016 Pedro Moncayo Community Health Survey (previously known as SADHS-PM) [39]. In 2022, a total of 505 participants completed a follow-up assessment in July–September. Participants were between 17 and 23 years of age and included 382 who were previously examined in 2016. A total of 119 individuals were newly recruited using SILC and through word of mouth. Additional details of the 2016 and 2022 examinations have been previously published [40,41].

The present analyses include 528 participants from the July–October 2016 examination and 487 participants from the July–September 2022 examinations who had all variables of interest. Longitudinal analyses using data from the 2016 and 2022 assessment periods included a total of 657 unique participants and 1012 observations.

### 2.3. Interviews and Examinations

Caregivers of the ESPINA participants completed surveys to obtain socioeconomic and demographic information in 2016, while study participants completed the survey in 2022. In the July–October examination in 2016 and the July–September examination in 2022, we examined participants in local schools when they were out of session. Examiners were blinded to participants’ exposure status. Height measurements were taken using a standard height board in accordance with established protocols, while weight was recorded with a digital scale (Tanita model 0108 MC; Tanita Corporation of America, Arlington Heights, IL, USA). Height-for-age and BMI-for-age z-scores were then computed based on the World Health Organization growth reference standards [42] for the 2016 assessment, and BMI for both time periods.

### 2.4. Measurement of Biomarkers

(A) Acetylcholinesterase activity (AChE) and hemoglobin concentration

AChE activity is used as a physiological marker for pesticide exposure [43], as OPs and carbamates inhibit the enzymatic activity of AChE and increase acetylcholine levels in the cholinergic synapse [9,32,44]. AChE activity and hemoglobin concentration were measured in fresh finger-stick capillary blood samples using the EQM-Test-mate ChE Cholinesterase Test System 400 during the examinations (EQM Research Inc., Cincinnati, OH, USA) in 2016 and 2022. AChE activity was measured on the same day that the urine samples were collected for pesticide analysis.

(B) Urinary pesticide biomarkers

*Urine samples.* Urine samples were collected at home upon awakening by the participants on the day of the study assessment. The samples were brought to the examination site in the morning by the participants, aliquoted, and then frozen on site at −20 °C. At the end of each day, the samples were transported to Quito for storage at −80 °C. The samples were then transported frozen using a specialized courier to UCSD, where they were stored at −80 °C. The samples were then shipped frozen from UCSD to the National Center for Environmental Health, Division of Laboratory Sciences of the CDC (Atlanta, GA, USA) for the quantification of 2,4-Dichlorophenoxyacetic acid (2,4-D), organophosphates, neonicotinoids, and pyrethroids, and to the Laboratory for Exposure Assessment and Development in Environmental Research at Emory University (Atlanta, GA, USA) for the quantification of creatinine, glyphosate, and bis-dithiocarbamate fungicides. To ensure the precision and credibility of the analytical results, standardized quality control and assurance procedures were implemented. Any study samples that did not meet the statistical quality control criteria were reprocessed through re-extraction [45,46].

*Urinary biomarkers of organophosphates, pyrethroids, and 2,4-D.* Pesticide urinary biomarkers quantified in 2016 and 2022 included organophosphates such as 3,5,6-trichloro-2-pyridinol (TCPy), a metabolite of chlorpyrifos and chlorpyrifos-methyl; PNP, a non-specific metabolite of parathion, methyl parathion, and related compounds; MDA, a metabolite of malathion; and 2-isopropyl-4-methyl-6-hydroxypyrimidine (IMPY), a metabolite of diazinon, pyrethroids, including the common metabolites 3-phenoxybenzoic acid (3-PBA), 4-fluoro-3-phenoxybenzoic acid (4-FP, a metabolite of cyfluthrin), and trans-3-(2,2-dichlorovinyl)-2,2-dimethylcyclopropane carboxylic acid (TCC, a metabolite of permethrin, cypermethrin, and cyfluthrin), and the phenoxy acid herbicide 2,4-D. Table S1 lists the pesticides measured in 2016 and 2022, along with their limits of detection (LODs), frequency of detection, and geometric means. Organophosphate and pyrethroid metabolites and 2,4-D were measured using liquid chromatography coupled with tandem mass spectrometry and isotope dilution in 1.0 mL of urine [47]. Study samples were analyzed in the same analytical run, together with calibrators, blanks, and quality control samples, to ensure data quality and reliability. The LODs were 0.60 µg/L for TCC, 0.5 µg/L for MDA, 0.1 µg/L for 3-PBA, IMPY, 4FP, TCPy, and PNP, and 0.15 μg/L for 2,4-D.

*Urinary neonicotinoid metabolites.* To measure neonicotinoid metabolite concentrations [i.e., 5-hydroxy imidacloprid (OHIM), acetamiprid-N-desmethyl (AND), imidacloprid (IMID), clothianidin (CLOT), acetamiprid (ACET), and thiacloprid (THIA)], targeted metabolites were quantified via the enzymatic hydrolysis of 0.5 mL of urine and online solid-phase extraction to release, extract, and concentrate the target biomarkers, followed by reversed-phase high-performance liquid chromatography–tandem mass spectrometry (HPLC–MS–MS/MS) via electrospray ionization [45]. The LODs were 0.40 µg/L for OHIM and IMID, 0.30 µg/L for ACET, 0.20 µg/L for AND, and CLOT, and 0.03 µg/L for THIA in 2016. The LODs were 0.10 µg/L for OHIM, 0.15 µg/L for AND, and 0.10 µg/L for CLOT in 2022.

*Urinary glyphosate concentration.* To analyze glyphosate, 250 μL urine samples were fortified with isotopically labeled glyphosate, brought to a final volume of 1 mL using doubly deionized water, and processed through C18 solid-phase extraction (SPE). The glyphosate was then derivatized into its heptafluorobutyl derivative and subsequently concentrated for measurement. Urine samples were randomized using a Fisher–Yates shuffling algorithm prior to analysis to reduce potential batch effects [45,48,49]. The concentrated extracts were examined using gas chromatography–mass spectrometry with electron impact ionization in multiple-ion monitoring mode. The LOD was 0.25 μg/L, with an RSD of 3%.

*Urinary ETU and PTU concentrations.* A volume of 800 µL of each urine sample was treated with isotopically labeled internal standards and 50 µL of 2.2 N hydrochloric acid. The prepared samples underwent extraction through Isolute solid-liquid extraction (SLE) cartridges. The eluates were evaporated to dryness and reconstituted with 100 µL of 0.1% formic acid. The final extracts were analyzed via liquid chromatography–mass spectrometry (LC/MS) equipped with electrospray ionization, employing the multiple-ion monitoring mode to quantify ETU and PTU along with their labeled counterparts [50]. Isotope dilution calibration was used for quantification, with detection limits of 0.625 ng/mL for ETU and 1.625 ng/mL for PTU, and a relative standard deviation (RSD) of 7%. Urine samples were randomized before analysis using the Fisher–Yates shuffle method to minimize the risk of batch-related biases during processing [45,48,49].

(C) Urinary creatinine concentration.

Urinary creatinine concentrations were measured using liquid chromatography electrospray ionization coupled with tandem mass spectrometry [51]. Urine specimens were randomized before analysis using the Fisher–Yates shuffling method to minimize potential batch-related bias.

(D) Quantitation of liver biomarkers

*ALT and AST concentration.* Liver function tests, including serum ALT and AST, were performed at US Specialty Labs, San Diego, California. A 0.4 mL serum sample was used to measure ALT and AST (kit number: Beckman Coulter: OSR6607 for ALT and OSR6609 for AST) levels using a spectrophotometric analytical principle with a fixed-point colorimetric method using a AU680 Clinical Chemistry Analyzer (Beckman Coulter, Brea, CA, USA). They were measured in duplicate, and the average of both values was used for the analyses.

*Quantitation of CK-18 (M65) and Caspase-Cleaved CK-18 (M30).* CK-18 M65 and M30 determination was conducted in 25 μL of plasma using the M30 Apoptosense^®^ ELISA (Cat no. 10011) and M65^®^ ELISA (Cat. No. 10020) kits, both of which were obtained from PEVIVA AB (Bromma, Sweden), following the manufacturer’s instructions. The plates were read at 450 nm, and data analysis was performed by using the cubic spline algorithm in GraphPad Prism 9 (Graph Pad Software Inc., La Jolla, CA, USA). CK-18 measures were conducted at the Feldstein Laboratory at UC San Diego.

### 2.5. Statistical Analysis

Means and standard deviations (SDs) or medians (25th–75th percentiles) for participant characteristics were calculated using descriptive statistics. The *p*-trend (*p*-value for trend) for participant characteristics across urinary metabolite concentrations was calculated using linear regression and modeling of the urinary metabolite concentration as a log-transformed continuous variable.

The present analyses include pesticide urinary metabolites that were detectable in at least 50% of the samples in a cross-sectional analysis in 2016 (2,4-D, TCPy, PNP, 3-PBA, glyphosate, ETU, and PTU) and 2022 (2,4-D, AND, TCPy, PNP, and 3-PBA). In longitudinal analyses, we included 2,4-D, AND, AChE, TCPy, PNP, and 3-PBA. Cross-sectional and longitudinal models were also conducted to look at the association between AChE concentration and all liver biomarkers. We imputed values below the LOD using multiple imputation. The multiple-imputation model was built as a log-linear “Tobit” regression model fitted on the basis of pesticide biomarker concentrations above the LOD [52]. The imputation of values was performed via a backward elimination procedure that started with the following 13 variables potentially associated with the urinary metabolite concentrations in the full model: age, sex, ethnicity, Z-BMI-for-age and Z-height-for-age, family income, cohabitation with agricultural workers, residential distance to the nearest flower crop, flower crop areas within 150 m of participants’ homes, sexual maturation, creatinine concentration, and TCPy and PNP concentrations (metabolites detected in all participants and potentially used together with other pesticides). After running the model, the variables with the highest *p*-values were eliminated from the imputation model in succession until all variables in the model reached a *p*-value threshold of <0.1. The final variables selected via backward selection were age, sex, race, creatinine level, and Z-BMI-for-age (Table S1).

The Tobit model was used to impute censored pesticide metabolite values, with imputed values rescaled so that the highest remained just below the limit of detection (LOD). This process was repeated across 1000 imputed datasets to generate multiple imputation estimates of the parameters for the primary model assessing pesticide–outcome associations [53]. Final parameter estimates and their variances were obtained by combining results from the 1000 datasets, incorporating both within- and between-imputation variability [53]. For the purposes of presenting the metabolite concentrations (Table S1), participants with values below the LOD were imputed using LOD/√2.

For each imputed dataset, GEE was used to model associations between urinary pesticide metabolites and liver biomarkers cross-sectionally and longitudinally (2016–2022). The 2016 model adjusted for age, sex, ethnicity, BMI for age z-score, and specific gravity. The 2022 and longitudinal GEE models were adjusted for the same covariates, apart from including BMI instead of BMI for age z-score.

We assessed the interactions of the main associations with sex and age using a multiplicative term in the GEE model for all pesticide metabolites (sex*log [pesticide metabolite] or age*log [pesticide metabolite]) and stratified analyses. When the interaction term was borderline significant (*p* < 0.10), the association was stratified by median splits of the effect modifier. For each metabolite, a quadratic term was added to metabolite models to test the presence of curvilinear associations with respect to the response variables. Time effects were also assessed by including an interaction term between the pesticide metabolite and assessment period in the GEE model.

Figures were created for associations with statistically significant interaction terms with age or sex, statistically significant curvilinear terms, or significant linear associations. We used locally estimated scatterplot smoothing (LOESS) to graph the observed relationships between 500 ranks of metabolite concentrations with liver markers, with a smoothness factor of 0.75. To account for the skewed distribution of metabolite concentrations and enhance the clarity of association plots, we applied a natural logarithmic transformation to the metabolite values on the x-axis. Each rank was then represented by the mean of the log-transformed concentrations.

Logistic regression was used to evaluate whether increasing urinary pesticide metabolite concentrations were associated with elevated liver enzymes (ALT or AST) in the 2022 ESPINA sample, adjusting for the same GEE covariates. For the 2016 cross-section, elevated concentrations were defined using age-appropriate adolescent cut-offs based on the Canadian Laboratory Initiative in Pediatric Reference Values: ALT > 27 U/L for males and >26 U/L for females, and AST > 38 U/L for males and >28 U/L for females. These thresholds reflect the upper bounds of the 95% confidence intervals for individuals aged 1 to <19 years [54]. For the 2022 cross-section, sex-specific adult thresholds were also based on the Canadian Health Measures Survey: ALT > 78 U/L for males and >41 U/L for females, and AST > 54 U/L for males and >34 U/L for females [55]. These reference values were appropriate for men aged 18–49 years and women aged 12–49 years. All metabolite concentrations were natural log-transformed to reduce skewness and approximate linearity. Odds ratios (ORs) represent the change in odds of elevated ALT or AST levels per 50% increase in each metabolite concentration.

The quantile g-computation method was applied to further investigate the 2016 cross-sectional associations between pesticide mixtures and liver biomarkers, using the R package “qgcomp” in R Studio. This approach quantifies the impact of collectively increasing all exposures by one quantile, enabling distinction between those with positive and negative relationships to the outcomes [56]. The assigned weights reflect the relative contribution of each exposure to the overall estimated effect. Using quantile g-computation, we tested the associations between a simultaneous 1 quartile increase in a mixture of 2,4-D, AND, TCPy, glyphosate, MDA, 3-PBA, PNP, ETU, and PTU concentrations (all naturally log-transformed) and liver concentrations, adjusting for the same covariates as the GEE models. To address potential false positive findings due to multiple comparisons, we applied a false discovery rate (FDR) correction using the Benjamini–Hochberg procedure to all *p*-values presented, including interaction terms. Analyses in this manuscript were conducted in R Studio 2024.04.0 for windows.

## 3. Results

### 3.1. Participant Characteristics

Table 1 shows the participant characteristics for the 2016 (N = 528) and 2022 (N = 487) ESPINA cycles. The mean age of participants was 14.5 years for the ESPINA 2016 examination and 20.3 years for the 2022 examination, with an almost equal distribution of males and females in both cycles. In 2016, Indigenous participants comprised 21.8% of the sample, while Mestizo participants accounted for 78.2%. In the 2022 cycle, Indigenous representation slightly increased to 22.4%, with Mestizo participants comprising 77.6%. The mean BMI-for-age Z-score in 2016 was 0.38, with a corresponding mean BMI of 20.94. By the 2022 follow-up, the mean BMI had increased to 23.96. A total of 67% and 64% of the participants lived with agricultural workers in 2016 and 2022, respectively. Participant characteristics across tertiles of 3-PBA are also presented in Table 1. Participants’ race, BMI-for-age z score, creatinine, and estimated specific gravity levels differed by urinary 3-PBA concentrations in 2016. It was noteworthy that Mestizo participants had higher 3-PBA concentrations. Only specific gravity differed across concentrations of 3-PBA in 2022.

The observed levels of liver biomarkers in our study participants in 2016 were largely within the expected range for their age group, with only about 1% exceeding the sex-specific upper bounds of the 95% confidence intervals (i.e., ALT: >27 for males, >26 for females; AST: >38 for males, >28 for females) previously reported for adolescents, indicating rare elevations [54]. However, in the 2022 assessment, using sex-specific adult thresholds [55], the prevalence of elevated liver enzymes was notably higher among females than males. Specifically, elevated AST was observed in 14.09% of females compared to 4.80% of males, while elevated ALT was present in 9.02% of females versus 2.80% of males.

### 3.2. Association Between Pesticide Metabolites and Liver Biomarkers

**Figure 1:**
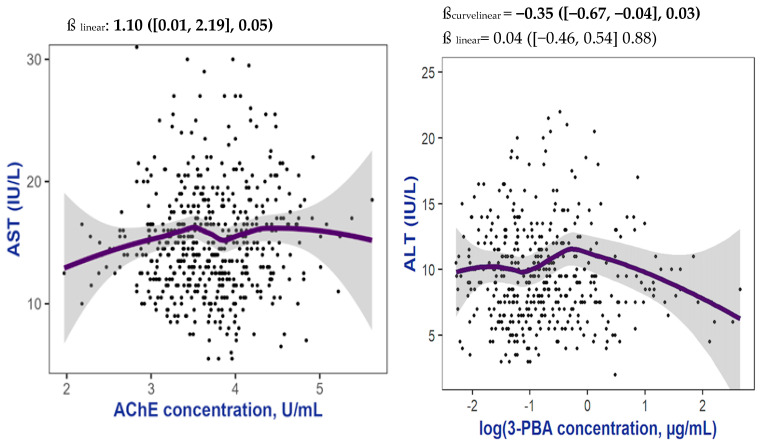
Associations of AChE activity with ALT concentrations and 3-PBA concentrations with ALT among adolescent participants in 2016 examined in the ESPINA study in Pedro Moncayo, Ecuador. Abbreviations: ALT: alanine aminotransferase, AST: aspartate aminotransferase, AChE: acetylcholinesterase, 3-PBA: 3-phenoxybenzoic acid. Adjusted for age, ethnicity, sex, creatinine, and Z-BMI-for-age. For analyses involving AChE, the models were further adjusted for hemoglobin. The solid lines represent the regression lines for each exposure–outcome pair, whereas the dashed lines indicate the 95% confidence intervals (CIs) around the point estimates, representing the range within which the true value is likely to fall with 95% confidence. The dots represent the observed data points. β_linear_ refers to the linear association between AST and AChE, adjusting for age, ethnicity, sex, urinary creatinine, Z-BMI-for-age, and hemoglobin in the case of AChE. β_curvilinear_ refers to the curvilinear association between ALT and 3-PBA, modeled with a quadratic term (i.e., 3-PBA × 3-PBA), and adjusted for the same covariates: age, ethnicity, sex, urinary creatinine, Z-BMI-for-age, and hemoglobin.

There were no consistent statistically significant associations between urinary pesticide biomarker levels and AST, ALT, CK-18 M30, and CK-18 M65 in cross-sectional analyses in 2016 (Table 2) or 2022, nor in longitudinal analyses. AChE was positively associated with all liver biomarkers in 2016 and 2022; however, it was borderline statistically significant only with AST in 2016 (Table 2, Figure 1). In the 2016 cross-section, a significant curvilinear association was observed between 3-PBA and ALT (p_quadratic_ = 0.03) (Table 2, Figure 1).

In 2016, there were statistically significant sex interactions between 3-PBA and ALT (β_pesticide*sex_ = 1.13 U/L [−0.00, 2.26], *p* = 0.05, Table 1), AST (β_pesticide*sex_ = 1.01 U/L [0.08, 1.93], *p* = 0.03), and CK18 M65 (β_pesticide*sex_ = 28.13 U/L [3.47, 52.80], *p* = 0.03). Sex-stratified analyses indicated a borderline positive association between 3-PBA levels and CK18 M65 among males (β = 4.20 U/L; 95% CI: −0.37, 8.78; *p* = 0.07), while a non-significant negative association was observed in females (β = −1.98; 95% CI: −6.94, 2.98; *p* = 0.43) (Figure S1). A similar pattern of non-significant positive associations in males was observed for ALT (β = 0.29; 95% CI: −0.13, 0.70; *p* = 0.18) and AST (β = 0.02; 95% CI: −0.28, 0.31; *p* = 0.91). In contrast, non-significant negative associations were seen in females for ALT (β = −0.07; 95% CI: −0.35, 0.21; *p* = 0.61) and AST (β = −0.21; 95% CI: −0.56, 0.14; *p* = 0.24) (Figure S1). However, 2022 cross-sectional (Table 3) and longitudinal analyses (Table 4) did not show significant interactions by sex.

Overall, we did not observe clear evidence of effect modification by age, with only two significant interaction terms in 2016 (PNP-ALT and 3-PBA-ALT associations, Table 2, Figure S2) and 2022 (2,4-D-AST and PNP-AST associations, Table 3, Figure S3). After applying FDR correction for multiple comparisons, none of the observed associations or interaction terms remained statistically significant. Longitudinal analyses did not show significant interactions by age and sex (Table 4).

### 3.3. Odds Ratios for Elevated AST and ALT Levels

Due to the low prevalence of elevated liver enzymes in the 2016 sample (<1%), odds ratios were estimated only for the 2022 cross-sectional data. Across all models, no statistically significant associations were observed between urinary pesticide biomarkers and elevated AST or ALT levels. For AST, the odds ratios (ORs) per 50% increase in metabolite concentrations were as follows: 2,4-D (OR = 1.03; 95% CI: 0.80–1.33), TCPy (OR = 1.03; 95% CI: 0.84–1.27), PNP (OR = 1.11; 95% CI: 0.83–1.47), 3-PBA (OR = 0.94; 95% CI: 0.77–1.14), AND (OR = 1.03; 95% CI: 0.93–1.14), and AChE (OR = 1.09; 95% CI: 0.63–1.88). For ALT, the corresponding ORs were: 2,4-D (OR = 1.03; 95% CI: 0.80–1.33), TCPy (OR = 1.04; 95% CI: 0.83–1.31), PNP (OR = 1.13; 95% CI: 0.82–1.56), 3-PBA (OR = 0.88; 95% CI: 0.70–1.11), AND (OR = 1.05; 95% CI: 0.94–1.17), and AChE (OR = 1.41; 95% CI: 0.99–2.00).

### 3.4. Pesticide Mixture Modeling

Using quantile g-computation, we observed no statistically significant associations between a one-quartile increase of the pesticide mixture with concentrations of any liver biomarkers evaluated in ESPINA 2016: ALT (β = 0.725, 95% CI: [−6.189, 7.638], *p* = 0.839), AST (β = 0.847, 95% CI: [−6.311, 8.004], *p* = 0.818), CK18-M30 (β = 1.184, 95% CI: [−40.837, 43.204], *p* = 0.956), and CK18-M65 (β = 19.472, 95% CI: [−36.228, 75.171], *p* = 0.498) and evaluated in ESPINA 2022: ALT (β = −0.285, 95% CI: [−169.811, 169.241], *p* = 0.997), AST (β = 0.275, 95% CI: [−121.742, 122.293], *p* = 0.996).

## 4. Discussion

This study, one of the largest to assess urinary pesticide metabolite levels and liver biomarkers in adolescents with follow-up into early adulthood, found no consistent associations between urinary pesticide metabolite levels, including 2,4-D, TCPy, PNP, 3-PBA, glyphosate, ETU, and PTU, and liver biomarkers (ALT, AST, and CK18-M65) in either cross-sectional or longitudinal analyses. Our findings, however, suggested differing associations by sex in the associations of 3-PBA with AST, ALT, and CK18-M65 in adolescence, but not in young adulthood. Other isolated findings emerged, such as a positive association of AChE activity with AST in adolescence but not in young adulthood, or curvilinear and age-specific associations of PNP, 3-PBA, and glyphosate with ALT in adolescence only. Although a few nominal associations were initially observed, they lacked consistency across time points and became non-significant after FDR correction, reinforcing the interpretation that these findings may reflect chance rather than true effects.

Although most associations were null, a borderline positive association was observed between AChE activity and AST levels. We observed that a greater AChE concentration, marking lower OP exposure, was associated with higher AST levels, which contradicts findings from other studies with inverse or null findings. For example, Patil et al. (2003) reported that decreased AChE activity was associated with increased ALT and AST levels among 85 male pesticide sprayers with 3–10 years of exposure [57]. In a longitudinal study of greenhouse workers, García-García et al. (2016) observed significantly lower erythrocyte AChE activity during both low- and high-exposure periods, but no differences in ALT or AST compared to controls [58]. Similarly, although not directly measuring AChE, Manfo et al. (2020) found a 28.4% elevation in ALT (*p* = 0.002) among pesticide-exposed farmers compared to non-occupational controls, although AST was unaffected [21]. Overall, while the majority of studies on occupational pesticide exposure report elevated levels of both aminotransferases (i.e., AST and ALT) [59] associated with pesticide exposure, some research has found no significant changes in ALT [29,60,61], AST [62], or both [63,64,65,66]. In occasional cases, findings have even shown a reduction in aminotransferase levels [61]. Additional studies are required to clarify these associations.

In the 2016 ESPINA cohort, elevated liver enzyme levels were rare, with only about 1% of participants exceeding the sex-specific upper confidence intervals previously established for adolescents, suggesting a low prevalence consistent with expected norms for that age group [67]. In contrast, the ESPINA 2022 cohort showed higher mean liver enzyme levels among adolescents and young adults when compared to U.S. populations. For example, the NHANES 1999–2004 study reported a lower mean (SD) ALT of 19.3 (15.6) U/L among adolescents aged 12–19 years, compared to 24.4 (17.3) U/L among adolescents and young adults in ESPINA 2022. Similarly, NHANES 2011–2018 data from U.S. adults (mean age of 47 years; range 32–61) showed a lower mean (range) ALT of 20.0 (15.0–27.0) U/L [67]. A similar trend was observed for AST levels: NHANES 1999–2004 adolescents had a mean (SD) AST of 22.8 (1.0) U/L, while ESPINA 2022 participants had a higher mean (SD) of 30.3 (12.4) U/L. Among U.S. adults in NHANES 2011–2018, AST levels also remained lower, with a mean (range) of 22.0 (18.0–26.0) U/L [68].

Although a few age-specific associations appeared to be statistically significant in the 2016 or 2022 cross-sectional analyses, these interactions were not consistent across cross-sectional or longitudinal models or across pesticide classes. We observed some evidence of consistent effect modification by sex in the associations between 3-PBA and the liver biomarkers AST, ALT, and CK18-M65. In stratified analyses, the associations did not reach statistical significance in either sex; however, the direction of the associations was generally positive in males and negative in females, suggesting potential sex-specific patterns. The observed sex-specific differences in the associations of 3-PBA with liver biomarkers in adolescents (positive non-statistically significant associations in males and negative non-significant associations in females) may reflect biological and hormonal factors that modulate susceptibility to pyrethroid exposure. Sex hormones such as estrogens and androgens influence hepatic enzyme activity, xenobiotic metabolism, and oxidative stress pathways, which are critical in the biotransformation and detoxification of pyrethroids [69,70]. Estrogens have been shown to upregulate antioxidant defenses and modulate cytochrome P450 enzyme expression, potentially conferring greater resilience to oxidative injury in females [71]. Pyrethroids additionally exhibit endocrine-disrupting properties, with evidence of androgen receptor antagonism and alterations in sex hormone levels that may differentially affect hepatic homeostasis in males and females [72,73]. Furthermore, sex differences in body fat distribution, plasma protein binding, and liver size may influence internal doses and kinetics of 3-PBA and its parent compounds, contributing to the divergent associations observed [70]. Together, these findings underscore the importance of considering sex as a biological variable in environmental health research, particularly when evaluating the hepatotoxic effects of endocrine-active pesticides such as pyrethroids.

Overall, we did not find significant relationships between pesticide biomarkers and liver biomarkers. It is plausible that for some pesticides, the amount of pesticide exposures in the time period when we conducted these exams (July–October) could have been too low to result in liver alterations. When comparing exposure levels between the ESPINA cohort and a representative sample of U.S. adolescents (NHANES) [74,75], we found that concentrations of some pesticide biomarkers were lower in ESPINA, while others were comparable or higher. For example, the geometric mean (GM) concentration of 3-PBA was 0.39 µg/g creatinine in 2016 and 0.51 µg/L (wet weight) in 2022 in ESPINA, compared to 0.54 µg/g creatinine in NHANES 2013–2014. These lower levels may partly explain the lack of associations with liver biomarkers. In contrast, TCPy concentrations were notably higher in ESPINA (3.07 µg/g creatinine in 2016 and 3.39 µg/L [ww] in 2022) than in NHANES (0.92 µg/g creatinine). Similarly, the levels of the neonicotinoid metabolite AND were higher in ESPINA (0.31 µg/g creatinine in 2016; 0.35 µg/L [ww] in 2022) compared to NHANES (0.18 µg/g creatinine). ESPINA study participants in 2016 had higher concentrations of urinary glyphosate than did a representative sample of adolescents in the U.S. from NHANES 2013–2014 (geometric means [GM]: ESPINA: 0.84 μg/g creatinine [0.74 μg/L [wet weight (ww)]] vs. 0.44 μg/g creatinine [0.41 μg/L (ww)]) [76]. Other exposures were comparable between ESPINA and NHANES. For example, PNP concentrations were 0.59 µg/g creatinine in ESPINA 2016, 0.63 µg/L (ww) in 2022, and 0.58 µg/g creatinine in NHANES. Likewise, 2,4-D concentrations were 0.27 µg/g creatinine in 2016 and 0.28 µg/L (ww) in 2022 in ESPINA, compared to 0.29 µg/g creatinine in NHANES [75]. Several pesticide biomarkers with detectability below 50% [77] have limited our ability to perform analyses with these pesticides. Nevertheless, the largely null findings—replicated across 2016, 2022, and longitudinal assessments—provide evidence that background-level exposures to the pesticides measured are unlikely to be associated with liver injury in this population.

This study offers several notable strengths. It is among the first to examine the relationships between pesticide biomarkers and liver function indicators within a prospective cohort followed from childhood into young adulthood. The sequential timing of sample urine collection has been demonstrated to affect exposure accuracy, and multiple urine collections are sometimes necessary to account for the intraindividual variation in urinary metabolites and to reflect a wider exposure window [78,79,80]. Our ability to measure urinary pesticide metabolites at two time points—both collected during the same season (summer)—enhances our estimation of participants’ typical exposure during that period. While additional measurements would have been ideal given the short half-lives of the pesticides assessed, these repeated measures still improve exposure characterization compared with a single time point. Another strength is the relatively large sample size within a population-based cohort across waves: 528 in 2016, 487 in 2022, and over 1000 observations in total for the longitudinal analysis. Additionally, the availability of urinary pesticide biomarkers in this community setting provides a unique opportunity to assess specific real-world exposures in a rural, agricultural population.

One limitation of our study is that some measured urinary organophosphate and pyrethroid metabolites, particularly TCPy, PNP, and 3-PBA, may reflect, in part, exposure to their preformed environmental degradation products, rather than direct exposure to the parent compounds through use in agricultural or household settings. In addition, urinary metabolites of these pesticides primarily provide information on a short window of exposure (~5 to 8 days) [81]. Nonetheless, because participants live in an agricultural community with year-round production enabled by Pedro Moncayo’s temperate, equatorial climate, participants in these settings are likely to have year-round background exposures. However, both biomarker assessments were conducted in summer, a season when flower production and pesticide use in local floriculture are generally lower, which may have led to lower measured concentrations than would be observed during peak application periods (October to May), which, in theory, would decrease inter-individual variability in exposure. However, pesticides with short biological half-lives, such as organophosphates and pyrethroids, show considerable fluctuations in urinary biomarker levels, with intra-individual variation often surpassing differences observed between individuals [82]. When a single urine sample is used to characterize typical exposure, this high within-person variability can result in exposure misclassification. Such misclassification is typically non-differential relative to the outcome, thereby biasing the estimated associations toward the null and potentially masking true effects. In contrast, glyphosate has a longer half-life in humans—ranging from about 3 to 14 days—and is mainly excreted unchanged in urine without significant bioaccumulation [83]. As a result, urinary glyphosate levels may better reflect subacute exposure compared to compounds such as ETU. Future studies should consider repeated sampling across multiple agricultural seasons to evaluate seasonal fluctuations in pesticide exposure and their impact on health outcomes.

Whereas urinary biomarkers of many pesticides reflect only short-term exposure windows, liver injury may develop over longer periods. This potential temporal misalignment between exposure and outcome could limit our ability to detect chronic liver effects in this study. However, some hepatotoxic exposures (e.g., medications or alcohol) can elicit acute changes in liver enzymes. For example, hydroxymethylglutaryl-CoA (HMG-CoA) reductase inhibitors (“statins”) are known to cause transient, asymptomatic elevations of serum aminotransferases [84]. Similarly, isoniazid (a first-line antitubercular drug) often induces mild, reversible increases in ALT and AST in a substantial fraction of patients (in the order of ~10–20%) [85]. These drug-induced enzyme elevations are generally benign and resolve even with continued therapy, illustrating how short-term hepatotoxic exposures can affect liver enzyme measurements. Our analysis is focused on identifying short-term hepatic responses to recent pesticide exposure. Moreover, the duration of exposure detection varies by chemical class, with shorter windows for organophosphates and pyrethroids and somewhat longer windows for certain herbicides and fungicides. Future studies should incorporate repeated biomarker measurements across time and seasons to more accurately characterize exposure patterns and potential chronic effects. Additionally, though we adjusted for key sociodemographic and behavioral covariates, residual confounding and unmeasured co-exposures to other measured pesticides cannot be fully ruled out.

Given the study’s 14-year follow-up period, we evaluated the potential for selection bias by comparing participant characteristics across the two follow-up waves used in this analysis compared to the baseline (Table S2). Although some differences were noted, most appeared consistent, with typical developmental trajectories from childhood into early adulthood, such as natural increases in hemoglobin levels and BMI. We observed a slight rise in the proportion of female participants over time (from 49.2% in 2008 to 50.5% in 2022), suggesting minimal sex-based differences in retention. Additionally, there was an uptick in the percentage of participants living with floricultural workers (from 57.2% to 70.7%). While this change may partly reflect differential follow-up, it likely also reflects the broader increase in floricultural production in Pedro Moncayo over time. Notably, greenhouse-based agriculture in the county expanded considerably during the study period, with the cultivated area increasing from 656 hectares (2% of the land area) in 2008 to 1495 hectares (4.47%) by 2016 [86]. This likely also led to a rise in employment in this sector, including among the family members of participants, which, in turn, may result in increased potential for pesticide exposures among participants. However, because the present analyses evaluate urinary pesticide metabolite concentrations over time, potential changes in exposure are accounted for in the longitudinal models. Overall, while some demographic and occupational patterns evolved over time, these changes do not appear to undermine the internal validity of our findings.

## 5. Conclusions

This study found no consistent associations between urinary pesticide metabolite concentrations (i.e., 2,4-D, TCPy, PNP, 3-PBA, glyphosate, ETU, and PTU) and liver biomarkers (ALT, AST, and CK18-M65) in either cross-sectional or longitudinal analyses. We observed some suggestive sex-specific associations between 3-PBA and AST, ALT, and CK18-M65 during adolescence (but not in young adulthood), but the associations were small and not statistically significant. The pesticide exposures observed in this population primarily reflected background-level exposures. As such, these findings do not rule out the possibility that liver alterations could occur at substantially higher exposure levels or in more vulnerable populations. Future studies evaluating populations with greater exposure intensity are needed to assess potential dose-dependent hepatotoxic effects more clearly.

## Figures and Tables

**Table 1:** Participant characteristics across tertiles of urinary 3-PBA concentrations among participants examined in the ESPINA study in Pedro Moncayo, Ecuador.

	Overall	3-PBA Tertiles ^			
		Lower	Middle	Higher	*p*-Trend
**ESPINA Jul-Oct 2016**					
Range (µg/L)	0.102–14.20	0.102–0.297	0.298–0.598	0.60–14.20	
N Participants	528	151	146	148	
Age (years)	14.46 (1.76)	14.63 (1.74)	14.15 (1.72)	14.56 (1.79)	0.453
Sex, Male	49.2%	53.0%	42.5%	51.4%	0.349
Ethnicity (Indigenous)	21.8%	26.5%	24.0%	14.2%	0.026
Ethnicity (Mestizo)	78.2%	73.5%	76.0%	85.8%	
Lived with an agricultural worker	334 (67.89%)	118 (71.95)	113 (68.90)	103 (62.80)	0.196
Body Mass Index (BMI), (kg/m^2^)	20.94 (2.95)	20.92 (2.93)	21.05 (3.18)	20.84 (2.74)	0.808
BMI-for-age Z Score	0.38 (0.86)	0.37 (0.82)	0.44 (0.89)	0.36 (0.90)	0.043
Creatinine (g/dL)	103.43 (64.20)	84.15 (49.47)	100.57 (56.72)	138.96 (70.26)	<0.001
Estimated Specific Gravity (mg/dL)	1.021 (0.006)	1.019 (0.005)	1.021 (0.005)	1.024 (0.005)	<0.001
Hemoglobin (g/dL)	12.95 (1.18)	13.00 (1.13)	12.88 (1.13)	13.04 (1.22)	0.220
ALT (U/L)	10.50 (6.64)	10.46 (6.25)	10.39 (6.20)	10.54 (8.23)	0.191
AST (U/L)	15.69 (6.25)	15.89 (6.08)	15.53 (5.18)	15.43 (7.74)	0.111
Cytokeratin 18 (M30) (U/L)	115.42 (409.95)	103.14 (113.14)	168.82 (770.55)	88.73 (61.26)	0.543
Cytokeratin 18 (M65) (U/L)	134.11 (158.09)	133.90 (121.82)	150.15 (258.29)	124.10 (81.95)	0.874
Acetylcholinesterase (U/mL)	3.70 (0.55)	3.74 (0.56)	3.69 (0.56)	3.70 (0.54)	0.137
**ESPINA Jul-Sep 2022**					
Range (µg/L)	0.071–14.00	0.071–0.34	0.35–0.67	0.68–14.00	
No. of Participants (N)	487	166	160	161	
Age (years)	20.31 (1.81)	20.31 (1.79)	20.26 (1.75)	20.36 (1.89)	0.695
Sex, % Male	50.5%	51.8%	46.2%	53.4%	0.816
Race (Indigenous)	22.4%	27.7%	21.2%	18.0%	0.960
Race (Mixed)	77.6%	72.3%	78.8%	82.0%	0.960
Lived with an agricultural worker	301 (63.91)	98 (61.64%)	105 (67.74%)	98 (62.42%)	0.273
BMI (kg/m^2^)	23.96 (3.47)	23.93 (3.61)	23.47 (3.29)	24.49 (3.45)	0.404
Specific Gravity (mg/dL)	1.019 (0.01)	1.016 (0.01)	1.019 (0.00)	1.022 (0.01)	<0.001
ALT (U/L)	24.42 (17.27)	25.95 (21.48)	22.64 (12.29)	24.62 (16.58)	0.453
AST (U/L)	30.26 (12.38)	30.93 (15.02)	29.34 (8.06)	30.50 (12.91)	0.837
Acetylcholinesterase (U/mL)	4.42 (0.65)	4.41 (0.62)	4.47 (0.65)	4.37 (0.67)	0.646

The values presented are percentages or means (SDs). Abbreviations: ALT: alanine aminotransferase, AST: aspartate aminotransferase, SD: standard deviation, BMI: body mass index, and 3-PBA: 3-phenoxybenzoic acid. ^ Nine participants with missing values for 3-PBA due to interfering substances were randomly assigned across the 3-PBA tertiles.

**Table 2:** Cross-sectional (2016) associations of urinary pesticide biomarker concentrations with liver biomarker concentrations in adolescents and young adults in the ESPINA study (N = 528).

Metabolites	Difference in Liver Biomarker Concentration per 50% Increase in Pesticide Biomarker Concentration ([95%CI], FDR-Corrected *p*-Value)			
ng/mL	ALT (U/L)	AST (U/L)	CK18 M30	CK18 M65
2,4-D	0.01 ([−0.31, 0.33], 0.95)	0.15 ([−0.18, 0.49], 0.78)	−10.96 ([−37.00, 15.09], 0.78)	1.55 ([−7.65, 10.75], 0.95)
AChE *	0.25 ([−0.82, 1.32], 0.92)	1.10 ([0.01, 2.19], 0.40)	31.19 ([−8.71, 71.10], 0.65)	15.38 ([−4.78, 35.53], 0.65)
TCPy	−0.01 ([−0.25, 0.22], 0.95)	0.01 ([−0.23, 0.25], 0.95)	−12.65 ([−33.52, 8.21], 0.71)	−5.37 ([−13.47, 2.74], 0.67)
PNP	−0.06 ([−0.43, 0.31], 0.95) ^a^	−0.12 ([−0.54, 0.29], 0.91)	−7.34 ([−23.87, 9.20], 0.78)	1.86 ([−7.36, 11.08], 0.95)
3-PBA	0.08 ([−0.16, 0.32], 0.90) ^1,±^	−0.07 ([−0.28, 0.15], 0.90) ^2^	−2.98 ([−8.50, 2.55], 0.75)	0.71 ([−2.84, 4.27], 0.95) ^3^
Glyphosate	0.10 ([−0.08, 0.28], 0.75) ^b^	0.06 ([−0.06, 0.18], 0.75)	4.21 ([−2.04, 10.47], 0.67)	2.05 ([−1.32, 5.43], 0.71)
ETU	−0.16 ([−0.43, 0.10], 0.71)	−0.12 ([−0.49, 0.25], 0.90)	−3.61 ([−7.94, 0.71], 0.60)	−3.40 ([−7.37, 0.57], 0.60)
PTU	0.11 ([−0.20, 0.41], 0.90)	0.24 ([−0.17, 0.65], 0.73)	−0.92 ([−3.87, 2.02], 0.90)	0.61 ([−3.05, 4.26], 0.95)

Abbreviations: 2,4-D: 2,4-Dichlorophenoxyacetic acid, AChE: acetylcholinesterase, TCPy: 3,5,6-trichloro-2-pyridinol, PNP: para-nitrophenol, 3-PBA: 3-phenoxybenzoic acid, ETU: ethylene thiourea, PTU: propylene thiourea, ALT: alanine aminotransferase, AST: aspartate aminotransferase, and CK18: soluble cytokeratin-18, CI: confidence Interval, FDR: false discovery rate. Adjustments: age, ethnicity, sex, specific gravity, and BMI-for-age z score. * Further adjusted for hemoglobin. ^1^ 3-PBA–sex interaction: ß_interaction_ = 1.13 ([−0.00, 2.26], 0.05/FDR-corrected *p*: 0.06). ^2^ 3-PBA–sex interaction: ß_interaction_ = 1.01 ([0.08, 1.93], 0.03/FDR-corrected *p*: 0.06). ^3^ 3-PBA–sex interaction: ß_interaction_ = 28.13 ([3.47, 52.80], 0.03/FDR-corrected *p*: 0.06). ^a^ PNP–age interaction: ß_interaction_ = −0.48 ([−0.96, −0.01], 0.04/FDR-corrected *p*: 0.06). ^b^ Glyphosate–age interaction: ß_interaction_ = −0.33 ([−0.67, 0.02], 0.06/FDR-corrected *p*: 0.06). ^±^ Curvilinear associations: β_quadratic_ = −0.35 ([−0.67, −0.04], 0.03/FDR-corrected *p*: 0.06).

**Table 3:** Cross-sectional (2022) associations of urinary pesticide biomarker concentrations with ALT and AST (N = 487) examined in the ESPINA study in Pedro Moncayo, Ecuador.

Metabolites	Difference in Liver Biomarker Concentration per 50% Increase in Pesticide Biomarker Concentration ([95%CI], FDR-Corrected *p*-Value)	
ng/mL	ALT (U/L)	AST (U/L)
2,4-D	−0.13 ([−0.88, 0.62], 0.95)	0.25 ([−0.36, 0.85], 0.84) ^a^
AChE *	1.83 ([−0.66, 4.32], 0.63)	0.82 ([−0.99, 2.63], 0.84)
AND	−0.08 ([−0.54, 0.38], 0.95)	−0.10 ([−0.43, 0.23], 0.88)
TCPy	−0.03 ([−0.79, 0.72], 0.95)	0.25 ([−0.32, 0.83], 0.84)
PNP	−0.16 ([−1.23, 0.92], 0.95)	0.37 ([−0.37, 1.10], 0.84) ^b^
3-PBA	−0.31 ([−1.21, 0.58], 0.86)	0.02 ([−0.76, 0.80], 0.95)

Abbreviations: 2,4-D:2,4-Dichlorophenoxyacetic acid, AChE: acetylcholinesterase, AND: Acetamiprid-N-desmethyl, TCPy: 3,5,6-trichloro-2-pyridinol, PNP: para-nitrophenol, 3-PBA: 3-phenoxybenzoic acid, ALT: alanine aminotransferase, AST: aspartate aminotransferase, CI: confidence interval, LOD: level of detection, FDR: false discovery rate. Adjustments: age, ethnicity, sex, specific gravity, and BMI. * Further adjusted for hemoglobin. ^a^ 2,4-D–age interaction: ß_interaction_ = 0.43 ([−0.01, 0.86], 0.05/FDR-corrected *p*: 0.05). ^b^ PNP–age interaction: ß_interaction_ = 0.42 ([0.00, 0.83], 0.05/FDR-corrected *p*: 0.05).

**Table 4:** Longitudinal associations (in 2016 and 2022) of urinary pesticide biomarker concentrations with ALT and AST. (N = 657, n_observations_ = 1012) were examined in the ESPINA study in Pedro Moncayo, Ecuador.

Metabolites	Difference in Liver Biomarker Concentration per 50% Increase in Pesticide Biomarkers ([95%CI], FDR-Corrected *p*-Value)	
ng/mL	ALT (U/L)	AST (U/L)
2,4-D	−0.06 ([−0.47, 0.35], 0.92)	0.23 ([−0.12, 0.58], 0.57)
AChE *	0.66 ([−0.74, 2.06], 0.72)	0.67 ([−0.35, 1.69], 0.57)
AND	0.01 ([−0.28, 0.30], 0.94)	0.02 ([−0.22, 0.26], 0.86)
TCPy	−0.05 ([−0.48, 0.38], 0.92)	0.18 ([−0.17, 0.52], 0.66)
3-PBA	−0.02 ([−0.39, 0.35], 0.94)	0.03 ([−0.32, 0.38], 0.86)
PNP	−0.10 ([−0.77, 0.57], 0.92)	0.19 ([−0.29, 0.67], 0.73)

Abbreviations: 2,4-D: 2,4-Dichlorophenoxyacetic acid, AChE: acetylcholinesterase, AND: acetamiprid-N-desmethyl, TCPy: 3,5,6-trichloro-2-pyridinol, 3-PBA: 3-phenoxybenzoic acid, PNP: para-nitrophenol, ALT: alanine aminotransferase, AST: aspartate aminotransferase, FDR: false discovery rate. Adjustments: age, sex, ethnicity, BMI, and specific gravity. * Further adjusted for hemoglobin.

## Data Availability

Data are available on request from the corresponding author due to privacy and ethical restrictions.

## Supplementary Materials

The following supporting information can be downloaded at: https://www.mdpi.com/article/10.3390/toxics13080685/s1, Table S1: Distribution of pesticide urinary metabolite concentrations with LODs, percentages detectable, geometric means and 95% confidence intervals measured from July–October 2016 in the ESPINA study in Pedro Moncayo, Ecuador. Figure S1: Sex-stratified analysis for adjusted associations between log-transformed urinary agrochemicals or metabolites and liver biomarkers or metabolites measured at the ESPINA 2016 in Pedro Moncayo, Ecuador. (A) 3-PBA and ALT. (B) 3-PBA & AST, and (C) 3-PBA and CK18 M65. Figure S2: Age-stratified analysis for adjusted associations between log-transformed urinary agrochemicals or metabolites and liver biomarkers or metabolites measured at the ESPINA 2016 in Pedro Moncayo, Ecuador. (A) PNP vs. ALT and (B) glyphosate vs. ALT. Figure S3: Age-stratified analysis for adjusted associations between log-transformed urinary agrochemicals or metabolites and liver biomarkers or metabolites measured at the ESPINA 2022 in Pedro Moncayo, Ecuador. (A) PNP vs. AST and (B) 2,4-D vs. AST. Table S2. Participant characteristics among all participants ever examined in the ESPINA study.
