# Supplementary material for: Pesticide and Liver Biomarkers Among Ecuadorian Adolescents and Adults Living in Agricultural Settings
Source: Toxics. 2025 Aug 18;13(8):685. doi: 10.3390/toxics13080685 (PMC12390044; doi:10.3390/toxics13080685)
Supplement: Supplementary file 1 [file toxics-13-00685-s001.zip › toxics-3716457-supplementary.pdf]

Pesticide and Liver Biomarkers Among Ecuadorian Adolescents and Adults Living in Agricultural Settings

Priyanka Mehta [1,†], Rajendra P. Parajuli [1,†], Briana N. C. Chronister [1], Kun Yang [1], Dana B. Barr [2], Xin M. Tu [1], Dolores Lopez-Paredes [3] and Jose R. Suarez-Lopez [1,*]

[1] Herbert Wertheim School of Public Health and Human Longevity Science, University of California San Diego (UCSD), La Jolla, CA 92093, USA; pmehta24@stanford.edu or priyanka@ucsd.edu (P.M.); rparajuli@health.ucsd.edu (R.P.P.); bnchronister@health.ucsd.edu (B.N.C.C.); yangkun123332@gmail.com (K.Y.); x2tu@health.ucsd.edu (X.M.T.)

[2] Gangarosa Department of Environmental Health, Rollins School of Public Health, Emory University, Atlanta, GA 30322, USA; dbbarr@emory.edu

[3] Fundación Cimas del Ecuador (CIMAS), De los Olivos E14-226 y, Quito 170124, Ecuador; dlopez@cimas.edu.ec

* Correspondence: jrsuarez@health.ucsd.edu; Tel.: +1-858-822-0165

† These authors contributed equally to this work as first authors.

Keywords: pesticide; insecticide and herbicide urinary metabolite; liver enzymes and biomarkers; adolescents; young adult; ESPINA study; Ecuador

Supplementary Table S1: Distribution of pesticide urinary metabolite concentrations with LODs, percentages detectable, geometric means and 95% confidence intervals measured from July–October 2016 in the ESPINA study in Pedro Moncayo, Ecuador.

Urinary pesticide biomarker	Class	LOD (µg/L)	Percent detectable	Geometric mean [95% CI]
**July October 2016 (adolescence)**				
**2,4-D**	Phenoxy acid herbicide	0.15	66.3%	0.353 [-3.644, 4.35]
**AND**	Neonicotinoid insecticide	0.2	36.6%	0.732 [-4.509, 5.973]
**TCPy**	Organophosphate insecticide	0.1	99.8%	2.688 [-1.661, 7.037]
**PNP**	Organophosphate insecticide	0.1	98.9%	0.516 [-2.976, 4.009]
**MDA**	Organophosphate insecticide	0.5	37.7%	0.898 [-1.951, 3.746]
**3-PBA**	Pyrethroid insecticide	0.1	84.3%	0.479 [-4.223, 5.181]
**Glyphosate**	Organophosphorus herbicide	0.25	100%	0.779 [-7.062, 8.620]
**July September 2022 (young adulthood)**				
**2,4-D**	Phenoxy acid herbicide	0.15	80.3%	0.354 [-3.634, 4.342]
**AND**	Neonicotinoid insecticide	0.05	67.6%	0.627 [-4.134, 5.388]
**TCPy**	Organophosphate insecticide	0.1	100.0%	3.385 [-0.975, 7.746]
**PNP**	Organophosphate insecticide	0.1	100.0%	0.630 [-3.026, 4.286]
**MDA**	Organophosphate insecticide	0.5	8.8%	0.828 [-2.728, 4.383]
**3-PBA**	Pyrethroid insecticide	0.1	98.8%	0.524 [-3.732, 4.780]

Abbreviations: 2,4-D:2,4-Dichlorophenoxyacetic acid, AND: Acetamiprid-N-desmethyl, TCPy: 3,5,6-trichloro-2-pyridinol, PNP: para-nitrophenol, MDA: malathion dicarboxylic acid, 3-PBA: 3-phenoxybenzoic acid

Supplementary Figure S1: Sex-stratified analysis for adjusted associations between log-transformed urinary agrochemicals or metabolites and liver biomarkers or metabolites measured at the ESPINA 2016 in Pedro Moncayo, Ecuador. (A) 3-PBA and ALT. (B) 3-PBA & AST, and (C) 3-PBA and CK18 M65

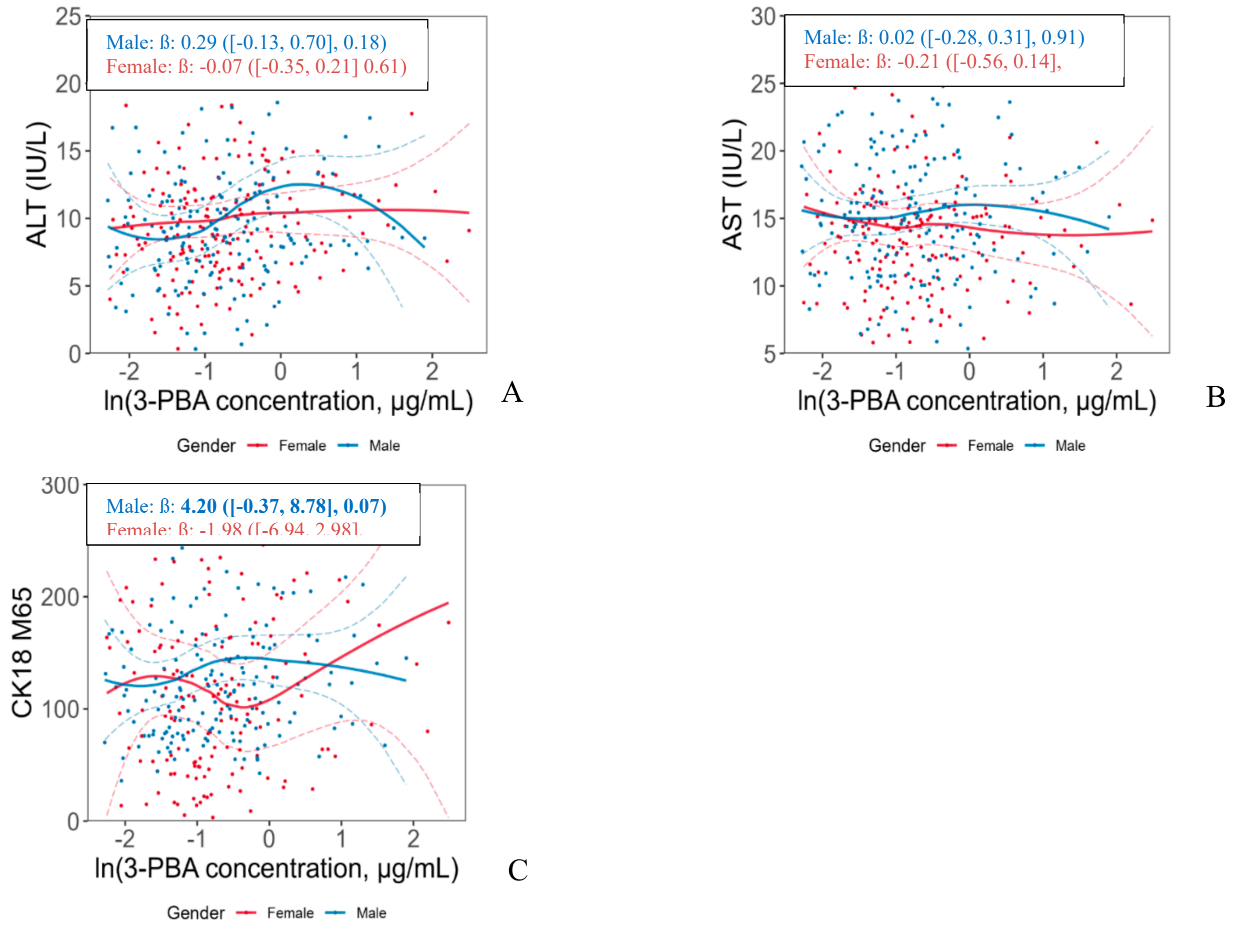

AST: aspartate aminotransferase, **ALT**: alanine aminotransferase, **CK18**: soluble cytokeratin-18, **3-PBA**: 3-phenoxybenzoic acid

Adjustments in the Model: Age, ethnicity, creatinine, and Z-BMI-for-age.

The solid lines in different colors represent the regression lines for each group, whereas the dashed lines of similar color indicate the 95% confidence intervals (CIs) around the point estimates, showing the range within which, the true value is likely to fall with 95% confidence.

Supplementary Figure S2: Age-stratified analysis for adjusted associations between log-transformed urinary agrochemicals or metabolites and liver biomarkers or metabolites measured at the ESPINA 2016 in Pedro Moncayo, Ecuador. (A) PNP vs. ALT and (B) glyphosate vs. ALT

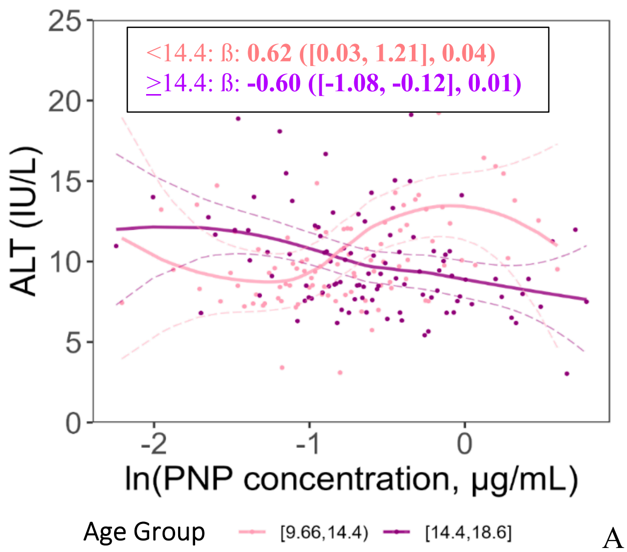

A

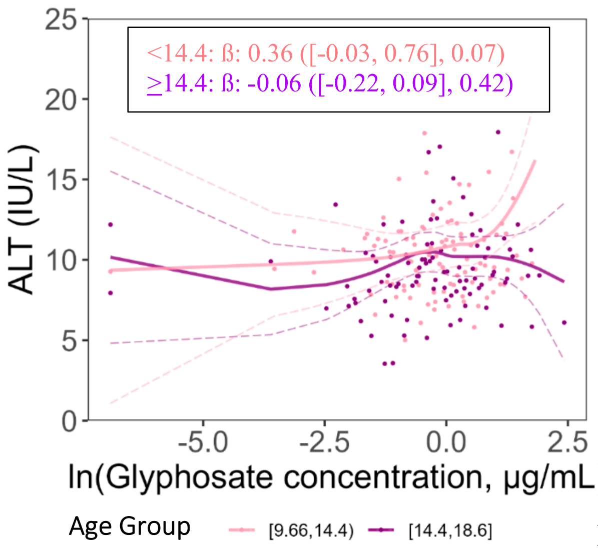

B

Adjustments in the Model: Age, ethnicity, sex, creatinine, and Z-BMI-for-age.

The solid lines in different colors represent the regression lines for each group, whereas the dashed lines of similar color indicate the 95% confidence intervals (CIs) around the point estimates, showing the range within which, the true value is likely to fall with 95% confidence.

ALT: alanine aminotransferase, **PNP**: para-nitrophenol

Supplementary Figure S3: Age-stratified analysis for adjusted associations between log-transformed urinary agrochemicals or metabolites and liver biomarkers or metabolites measured at the ESPINA 2022 in Pedro Moncayo, Ecuador. (A) PNP vs. AST and (B) 2,4-D vs. AST

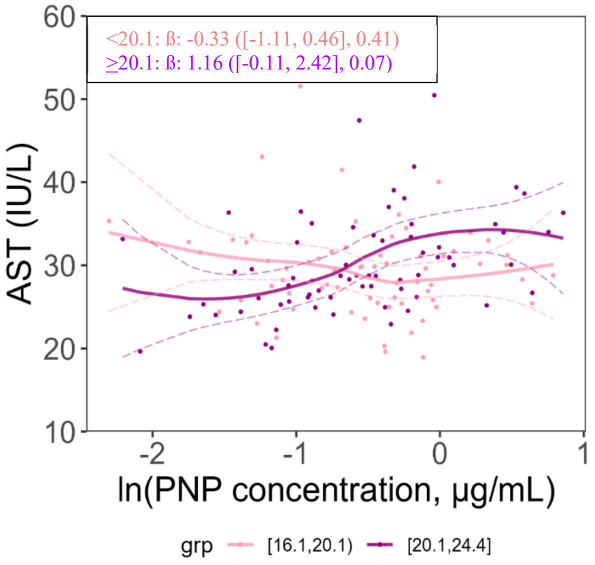

A

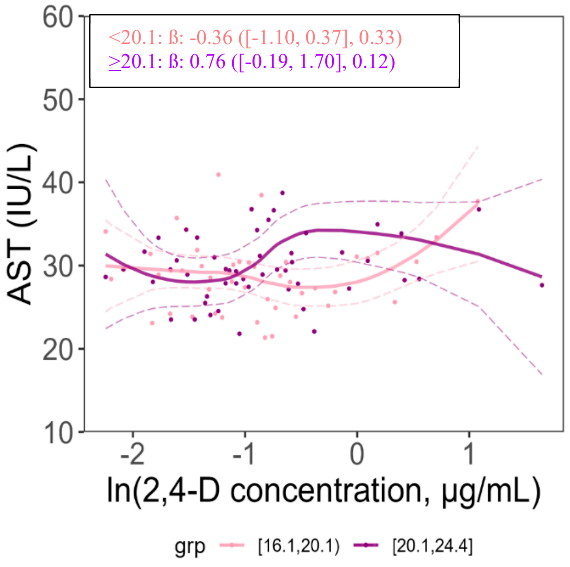

B

Adjustments in the Model: Age, ethnicity, sex, specific gravity, and BMI.

The solid lines in different colors represent the regression lines for each group, whereas the dashed lines of similar color indicate the 95% confidence intervals (CIs) around the point estimates, showing the range within which, the true value is likely to fall with 95% confidence.

AST: aspartate aminotransferase, **2,4-D**:2,4-Dichlorophenoxyacetic acid, **PNP**: para-nitrophenol

Supplementary Table S2. Participant characteristics among all participants ever examined in the ESPINA study.

	Jul-Aug 2008 (N=313)	Jul-Oct 2016 (N=535)	Jul-Oct 2022 (N=505)
**Age, years**	6.6 (1.6)	14.5 (1.8)	20.3 (1.8)
**Sex, %**			
Female	49.2%	50.8%	50.5%
Male	50.8%	49.2%	49.5%
**Ethnicity, %**			
Indigenous	21.1%	21.8%	18.6%
Mestizo	77.0%	77.9%	80.8%
**Lived with an agricultural worker**			
No	133 (42.8%)	175 (32.8%)	147 (29.3%)
Yes	178 (57.2%)	358 (67.2%)	354 (70.7%)
**Height, cm**	112.1 (10.3)	150.6 (9.4)	159.2 (8.7)
**BMI, kg/m^2**	16.1 (1.3)	20.9 (2.9)	24.0 (3.5)
**AChE, U/mL**	3.1 (0.5)	3.7 (0.5)	4.4 (0.6)
**Hemoglobin, g/dL**	12.6 (1.2)	13.0 (1.2)	14.7 (1.8)

Values presented are Mean (SD) or percent
